# To stay or not to stay intact as an allergen: the endolysosomal degradation assay used as tool to analyze protein immunogenicity and *T* cell epitopes

**DOI:** 10.3389/falgy.2024.1440360

**Published:** 2024-07-12

**Authors:** Elif Öztemiz Topcu, Gabriele Gadermaier

**Affiliations:** Department of Biosciences and Medical Biology, Paris Lodron University Salzburg, Salzburg, Austria

**Keywords:** endolysosomal degradation assay, antigen processing, allergens, *T* cell epitopes, mass spectrometry, immunogenicity, proteases

## Abstract

Antigen uptake and processing of exogenous proteins is critical for adaptive immunity, particularly for T helper cell activation. Proteins undergo distinct proteolytic processing in endolysosomal compartments of antigen-presenting cells. The resulting peptides are presented on MHC class II molecules and specifically recognized by *T* cells. The *in vitro* endolysosomal degradation assay mimics antigen processing by incubating a protein of interest with a protease cocktail derived from the endolysosomal compartments of antigen presenting cells. The kinetics of protein degradation is monitored by gel electrophoresis and allows calculation of a protein's half-life and thus endolysosomal stability. Processed peptides are analyzed by mass spectrometry and abundant peptide clusters are shown to harbor *T* cell epitopes. The endolysosomal degradation assay has been widely used to study allergens, which are IgE-binding proteins involved in type I hypersensitivity. In this review article, we provide the first comprehensive overview of the endolysosomal degradation of 29 isoallergens and variants originating from the PR-10, Ole e 1-like, pectate lyase, defensin polyproline-linked, non-specific lipid transfer, mite group 1, 2, and 5, and tropomyosin protein families. The assay method is described in detail and suggestions for improved standardization and reproducibility are provided. The current hypothesis implies that proteins with high endolysosomal stability can induce an efficient immune response, whereas highly unstable proteins are degraded early during antigen processing and therefore not efficient for MHC II peptide presentation. To validate this concept, systematic analyses of high and low allergenic representatives of protein families should be investigated. In addition to purified molecules, allergen extracts should be degraded to analyze potential matrix effects and gastrointestinal proteolysis of food allergens. In conclusion, individual protein susceptibility and peptides obtained from the endolysosomal degradation assay are powerful tools for understanding protein immunogenicity and *T* cell reactivity. Systematic studies and linkage with *in vivo* sensitization data will allow the establishment of (machine-learning) tools to aid prediction of immunogenicity and allergenicity. The orthogonal method could in the future be used for risk assessment of novel foods and in the generation of protein-based immunotherapeutics.

## Introduction

Antigen processing of exogenous proteins plays a fundamental role in adaptive immunity particularly in T helper (Th) cell activation. Upon antigen uptake, proteins are proteolytically processed and cleaved within the endolysosomal compartments of antigen-presenting cells (APCs). The resulting antigen-specific peptides are loaded onto MHC class II and presented to Th cells. The peptides, specifically recognized by *T* cells, are called *T* cell epitopes, and detailed knowledge of these epitopes helps to understand fundamental steps in *T* cell immunity (1). *T* cell epitopes can be determined experimentally by peptide mapping which requires antigen-specific human *T* cells and a large set of overlapping peptides sequences. Another method is *in silico* prediction which allow analysis of protein sequences (2–4), however these methods are limited to presentation on specific HLA types and always require experimental verification.

In 2005, the *in vitro* endolysosomal degradation assay was established to determine the susceptibility of proteins to endolysosomal proteases obtained from antigen-presenting cells (5). This assay allows determination of a protein's half-life and later, a refined version of the assay also monitored proteolytic peptides containing potential *T* cell epitopes (6). The endolysosomal degradation assay was mainly used for the analysis of IgE-binding molecules, which play a major role in type I hypersensitivity, affecting approximately 25%–30% of the population (7). Knowledge on the susceptibility to endolysosomal degradation and *T* cell epitopes will support the development of novel allergy diagnostics and therapeutics. To date, studies have mainly focused on purified allergens while degradation studies on more complex mixtures or allergen extracts are not available. For a few purified allergens, systematic comparisons of endolysosomal degradation and immunogenicity were performed.

In this review, we provide a comprehensive overview of studies using *in vitro* processing of allergens by the endolysosomal degradation assay. So far, 29 allergenic molecules (including isoallergens and variants) from different protein families, i.e., Bet v 1, Ole e 1-like proteins, pectate lyases, defensin polyproline-linked proteins, non-specific lipid transfer proteins, mite group 1, 2, and 5 allergens, and tropomyosins have been investigated. Furthermore, the methodological part of the assay is discussed in detail and specific improvements for the next level of use are suggested. In addition to proteolytic susceptibility, protein-specific features such as thermal stability, tertiary fold and isoelectric point are considered for the first time. This could help in establishment of more general concepts linking endolysosomal degradation and immunogenicity of allergens.

## Antigen processing of exogenous proteins and presentation on MHC II

### Antigen-presenting cells (APCs) and antigen uptake

APCs sample the extracellular milieu and capture exogenous antigens. Dendritic cells (DCs), B cells, and macrophages are considered professional APCs with different functions during an immune response. DCs and B cells internalize antigens for presentation on MHC II, with DCs acting as initiators of an immune response as they survey the periphery to capture antigenic substances for transport to secondary lymphoid organs. Antigen presentation on B cells contributes to the humoral immune response, whereas macrophages are primarily involved in pathogen clearance (8, 9). DCs are the most important APCs due to their effective antigen capture and survival, high MHC II levels, adhesion and costimulatory molecules (5, 9, 10). Antigen uptake occurs via macropinocytosis or clathrin-mediated endocytosis. Macropinocytosis is fluid phase endocytosis mediated by membrane invagination to form a vessel with a large volume of extracellular fluid. In DCs, this process is constitutive and allows the uptake of large volumes of fluid, in contrast to growth factor-driven macropinocytosis in macrophages. Clathrin-mediated endocytosis provided by cell surface receptors represents specific uptake by receptors of the C-type lectin family such as mannose and transferrin receptors, as well as Fc receptors (1, 11).

### Antigen processing by endolysosomal proteases

Internalized proteins are processed into peptides in multivesicular endolysosomal compartments. Proteins are initially translocated through vesicles referred to as early and late endosomes (9). Maturation of endosomes from early to late stages involves luminal acidification of vesicles to provide an optimal low pH environment for resident proteases. Early endosomes have a pH milieu ranging from 5.9–6.8 and late endosomes from 4.9–6.0 (12). Fusion of late endosomes with lysosomes is unidirectional and new hybrid organelles called endolysosomes are formed (13). Lysosomal proteins and hydrolases are abundant in this compartment, where internalized proteins are efficiently degraded into peptides (14).

### Antigen presentation on MHC II

Due to high allelic polymorphisms and thus amino acid variations in the binding region, MHC can bind a wide range of processed peptides with high affinity. Peptides from endolysosomal degradation are transported on MHC II molecules and presented to CD4^+^ *T* cells, while intracellular peptides obtained from proteasomal degradation are transported on MHC I and presented to CD8^+^ *T* cells (1). All nucleated cells express MHC I molecules, but only professional APCs constitutively express MHC II for surveillance of exogenous antigens (8). MHC II molecules are assembled in the endoplasmic reticulum, and contain an invariant chain, that facilitates translocation to the endosomes and protects the peptide-binding groove. MHC II maturation is completed after processing the invariant chain into the class II-associated invariant chain peptide (CLIP). CLIP is then replaced by high-affinity peptides of optimal length of 18–20 residues obtained by progressive proteolysis in endolysosomes. The MHC II peptide complex is finally inserted into the cell membrane (1, 15, 16). Notably, antigen uptake enhances MHC II synthesis in the endolysosomes of immature DCs. In contrast, mature DCs typically exhibit high levels of peptide-loaded MHC II molecules on their cell surface (15).

MHC II-bound peptides are recognized by CD4^+^ *T* cells via *T* cell receptors. The first interaction between naïve *T* cells and MHC II peptide complexes occurs at secondary lymphoid organs and results in activation and clonal expansion of antigen-specific effector CD4^+^ *T* cells or memory *T* cells. CD4+ *T* cells differentiate into Th2 subsets upon interaction with allergen-derived peptide-MHC II complexes combined with other interactions between the APC and *T* cell (e.g., CD40-CD40L, B7-CD28). Th2 cell differentiation is mainly driven by IL-4, low dose of antigen and low affinity between antigen and TCR (17–20). Consequently, Th2 cell differentiation also depends on protein stability during endolysosomal degradation, as optimal peptide abundance in late endosomes is a key factor for antigen presentation, *T* cell activation, and Th2 cell differentiation (19). Differentiated Th2 cells secrete cytokines, mainly IL-4, IL-5 and IL-13, which contribute to IgE class switch as well as mast cell and eosinophil activation (21, 22).

### Identification of *T* cell epitopes and simulated antigen processing methods

Processed peptides that are recognized by *T* cells and elicit an immune response are referred to as *T* cell epitopes. Peptides presented on MHC II are typically between 11 and more than 20 amino acids in length (23). *T* cell epitopes can be identified by *T* cell activation assays using short overlapping synthetic peptides covering the entire protein sequence. *T* cell activation is evaluated by assessing cell proliferation, expression of activation markers, or the production of effector cytokines (24). For *T* cell epitope mapping, peripheral blood mononuclear cells (PBMC) from allergic donors or allergen-specific *T* cell lines or clones were used. Using an array of synthetic overlapping peptides, stimulation indices of individual peptides reveal the allergen-specific *T* cell epitopes of allergens (25, 26). Another method to identify *T* cell epitopes is the elution of MHC II-bound peptides from APC and subsequent analysis by mass spectrometry (27). A high-throughput platform for the analysis of MHC II peptide binding has been established for SARS-CoV-2 and may be extended to other diseases (28). A comprehensive overview of identified *T* cell epitopes is provided by the Immune Epitope Database (www.iedb.org). In addition, *in silico* prediction tools can be used to reveal potential *T* cell epitopes (3, 4), which are however typically linked and thus restricted to individual human leukocyte antigens (HLAs).

Endolysosomal processing within APCs, including phagosomal activity, antigen degradation and presentation, can be measured by coupling labeled proteins to latex beads and monitoring intracellular and phagolysosomal degradation in a time-dependent manner by cytofluorometry. Ovalbumin, ß-lactoglobulin and peanut allergens have been studied using this intracellular degradation method (29–31). The results suggest a partial relationship between protein degradation and antigenicity, but further intracellular studies are required (31). An alternative method is the endolysosomal degradation assay which is straightforward and additionally allows identification of proteolytic peptides. It mimics antigen processing by incubating proteins with endolysosomal proteases derived from APCs (5). This assay does not require protein labeling or coupling but allows direct determination of proteolytic susceptibility and analysis of proteolytic peptides. This review focuses on endolysosomal degradation of allergens, which are IgE-binding proteins involved in allergic diseases that affect around 25%–30% of the population (7, 32)

## Antigen processing of allergens

### Endolysosomal degradation assay

**Figure 1:**
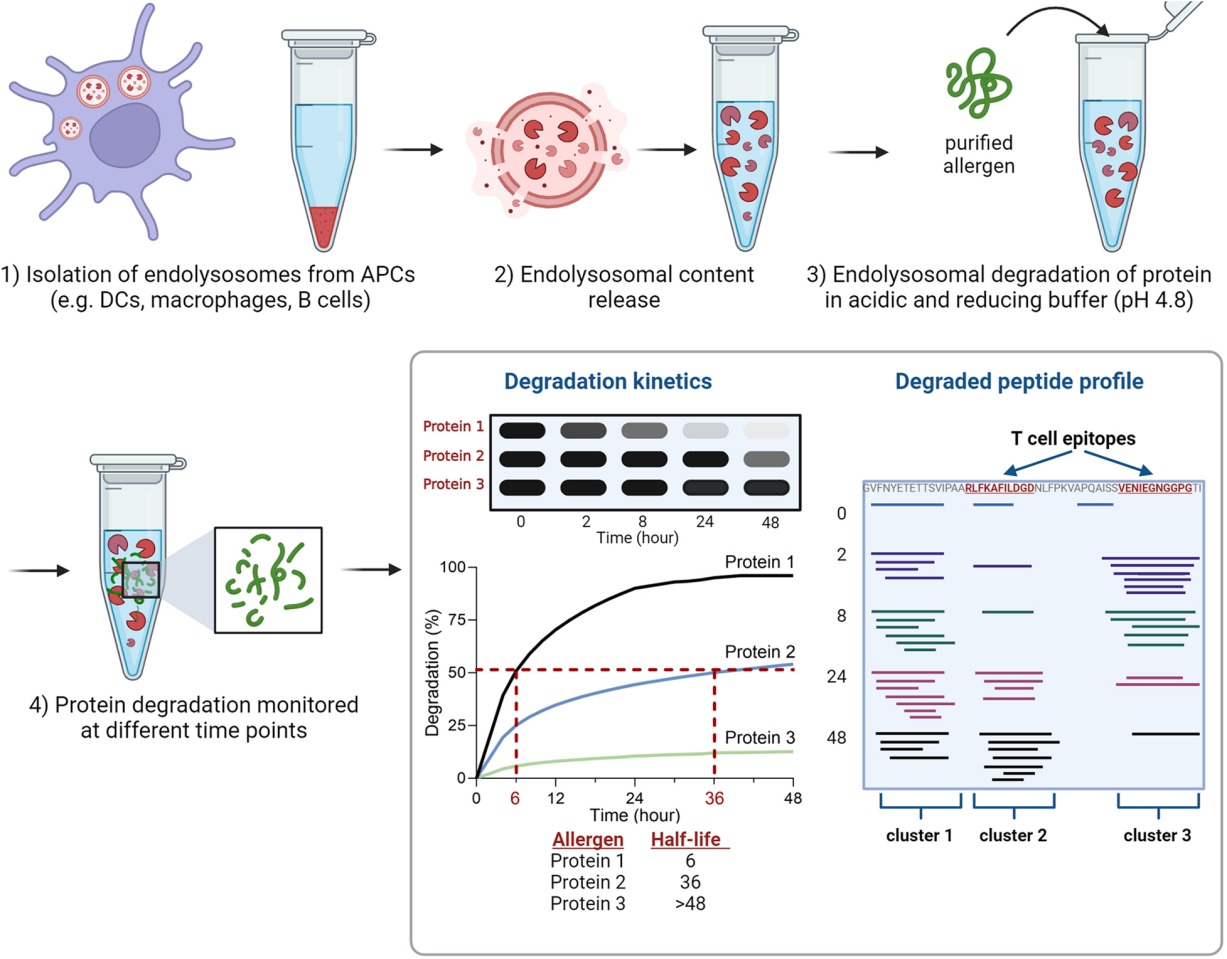
Overview of the experimental setup of the endolysosomal degradation assay. Analyses of protein degradation using gel electrophoresis enables the determination of the protein's half-life, while measurement of peptides using mass spectrometry identifies peptide clusters which include *T* cell epitopes.

The endolysosomal degradation assay is an *in vitro* technique used to study the susceptibility of proteins to endolysosomal proteases (Figure 1). It was introduced by Delamarre et al. and further refined by Egger et al. to monitor allergen degradation (5, 6). The endolysosomal proteases are obtained from cultivated APCs. The mouse DC line JAWS II has been predominantly used for this purpose, but monocyte-derived DCs, bone marrow-derived DCs, B cells and macrophages have also been investigated (6, 27, 33–37). After culturing APCs, the cells are lysed in Tris/acetate pH 7.0 with sucrose and the microsomes are isolated by differential centrifugation. To isolate the protein content consisting of endolysosomal proteins including proteases, the microsomes are subjected to repeated freezing in liquid nitrogen and thawing at room temperature. The obtained microsomal content includes endolysosomal proteins and proteases. To simulate endolysosomal degradation, the (purified) protein of interest is incubated with the endolysosomal proteins in an acidic buffer. Most studies incubated 5 μg of protein with 7 or 7.5 μg of isolated microsomal proteins. Typically, the assay is performed in pH 4.8 citrate buffer containing the reducing agent dithiothreitol to mimic endolysosomal conditions. In addition, digests at pH 5.9, 5.2 and 4.5 have been used to simulate early and late endosomal proteolysis, respectively (6, 27, 33–37). The degradation of the protein of interest is monitored over time (up to 72 h) using reducing SDS-PAGE gel analysis. The susceptibility to proteases can be determined by densitometric measurement and calculation of the half-life of the protein (Figure 1). It is worth mentioning, that the simulated *in vitro* degradation process is considerably slower compared to *in vivo* processing. This is due to significantly lower protease concentrations (10^6^-fold lower) used in the assay, which allows efficient protease usage and quantitative monitoring of the degradation (6). In this way, half-lives of the allergens can be calculated and used for comparison.

The proteolytic peptides are further analyzed by mass spectrometry (MS) to identify peptide clusters. Peptide clusters are defined as part of the protein sequence in which several overlapping peptide fragments are identified during time-dependent degradation (Figure 1). These peptide clusters can identify potential peptide candidates for presentation on MHC molecules (*T* cell epitopes). The analysis of mass spectrometry data allows the investigation of experimentally determined *T* cell epitopes within the identified clusters (6, 27, 33–37). The endolysosomal degradation assay showed that the stability of proteins against endolysosomal proteases of APCs can be related to peptide processing and thus to the immune response (33, 36, 38, 39).

### Different antigen presenting cells used for endolysosomal degradation of allergens

Originally, primary DCs from human (allergic) donors or mice were used to obtain endolysosomal proteases. Later, commercially available antigen presenting cell lines, such as the mouse cell line JAWS II were often used (Table 1). Compared to primary APCs, cell lines are an easily accessible and robust source, can be cultured in large quantities in a short time, and avoid the involvement of humans or animals. Comparison of human monocyte-derived DCs (mDCs), mouse bone marrow-derived DCs (BMDCs) and JAWS II showed that cathepsin A, B, C, D, L, S and Z, lysosomal prolylcarboxypeptidase and tripeptidyl peptidase 1 are present in all three DCs (6, 50). In terms of biological protease activity, similar half-lives of the major birch pollen allergen Bet v 1 were found after incubation with human and mouse primary DCs or the mouse cell line JAWS II and highly similar peptide clusters were generated. However, peptide appearance during the digestion and thus kinetics showed slight differences between the tested DCs. For example, after 36 h, peptides within the Bet v 1 residues_1–22_ were generated only by JAWS II, whereas the cluster at residue_21–55_ showed a slightly delayed appearance using human mDCs and JAWS II proteases (6). These results overall indicate that proteases from different DCs irrespective of their source have similar proteolytic activities and peptide profiles thus supporting the use of cell lines for the endolysosomal degradation assay (Table 1).

**Table 1:** Characteristics and half-lives of allergens analyzed by the endolysosomal degradation assay.

Allergen characteristics							Endolysosomal degradation				
Allergen molecule	Allergen source		Allergen tissue	Exposure route	Protein family/function	Mass of mature protein (kDa)	pI of mature protein	APCs used for isolation of endolysosomal enzymes	Buffer pH for digestion	Half-life (h)	References
rBet v 1.0101	Birch	*Betula verrucosa*	Pollen	Inhalant	Bet v 1-like, PR-10	17	5.4	Human mDCs	4.8	*5*	(27)
rBet v 1.0101	Birch	*Betula verrucosa*	Pollen	Inhalant	Bet v 1-like, PR-10	17	5.4	Human mDCs	4.8	2	(6)
rBet v 1.0101	Birch	*Betula verrucosa*	Pollen	Inhalant	Bet v 1-like, PR-10	17	5.4	Mouse BMDCs	4.8	2	(6)
rBet v 1.0101	Birch	*Betula verrucosa*	Pollen	Inhalant	Bet v 1-like, PR-10	17	5.4	JAWS II	4.8	2	(6)
rBet v 1.0101	Birch	*Betula verrucosa*	Pollen	Inhalant	Bet v 1-like, PR-10	17	5.4	JAWS II	4.8	*7*	(40)
rBet v 1.0101	Birch	*Betula verrucosa*	Pollen	Inhalant	Bet v 1-like, PR-10	17	5.4	JAWS II	4.8	*5*	(35)
rBet v 1.0101	Birch	*Betula verrucosa*	Pollen	Inhalant	Bet v 1-like, PR-10	17	5.4	A20	4.8	*>72*	(35)
rBet v 1.0101	Birch	*Betula verrucosa*	Pollen	Inhalant	Bet v 1-like, PR-10	17	5.4	RAW 264.7	4.8	*36*	(35)
rBet v 1.0101	Birch	*Betula verrucosa*	Pollen	Inhalant	Bet v 1-like, PR-10	17	5.4	Activated RAW 264.7	4.8	*>72*	(35)
rBet v 1.0101	Birch	*Betula verrucosa*	Pollen	Inhalant	Bet v 1-like, PR-10	17	5.4	Mouse BMDCs	4.8	*6*	(41)
rBet v 1.0101	Birch	*Betula verrucosa*	Pollen	Inhalant	Bet v 1-like, PR-10	17	5.4	JAWS II	4.8	*16*	(42)
rBet v 1.0101	Birch	*Betula verrucosa*	Pollen	Inhalant	Bet v 1-like, PR-10	17	5.4	JAWS II	4.8	*14*	(43)
rBet v 1.0201	Birch	*Betula verrucosa*	Pollen	Inhalant	Bet v 1-like, PR-10	17	5.8	Human mDCs	4.8	4	(6)
rBet v 1.0201	Birch	*Betula verrucosa*	Pollen	Inhalant	Bet v 1-like, PR-10	17	5.8	Mouse BMDCs	4.8	3	(6)
rBet v 1.0201	Birch	*Betula verrucosa*	Pollen	Inhalant	Bet v 1-like, PR-10	17	5.8	JAWS II	4.8	4	(6)
rBet v 1.0101 codon harmonized	Birch	*Betula verrucosa*	Engineered	–	Bet v 1-like, PR-10	17	5.4	JAWS II	4.8	*8*	(40)
rBM4 (hypoallergenic Bet v 1 fold variant)	Birch pollen	*Betula verrucosa*	Engineered	–	Bet v 1-like, PR-10	17	5.6	Human mDCs	4.8	*0.5*	(44)
rBM4 (hypoallergenic Bet v 1 fold variant)	Birch pollen	*Betula verrucosa*	Engineered	–	Bet v 1-like, PR-10	17	5.6	Mouse BMDCs	4.8	*9*	(41)
rOle e 1.0101 (A99 V, K106l, N111Q)	Olive	*Olea europaea*	Pollen	Inhalant	Ole e 1-like	16	6.2	JAWS II	4.8	*7*	(34)
rFra e 1.0101	European ash	*Fraxinus excelsior*	Pollen	Inhalant	Ole e 1-like	16	5.9	JAWS II	4.8	*20*	(34)
rSal k 5.0101 (K3N, G84D, I91 V)	Prickly saltwort	*Salsola kali*	Pollen	Inhalant	Ole e 1-like	16	5.0	JAWS II	4.8	*55*	(34)
rChe a 1.0101	Lamb's quarters, goosefoot	*Chenopodium album*	Pollen	Inhalant	Ole e 1-like	18	4.9	JAWS II	4.8	*65*	(34)
rPhl p 11.0101 (N24Q)	Timothy grass	*Phleum pratense*	Pollen	Inhalant	Ole e 1-like	16	5.0	JAWS II	4.8	*1*	(34)
rPla l 1.0101	English plantain	*Plantago lanceolata*	Pollen	Inhalent	Ole e 1-like	15	7.6	JAWS II	4.8	*>72*	(34)
rAmb a 1.0301	Short ragweed	*Ambrosia artemisiifolia*	Pollen	Inhalant	Pectate lyase	40	5.4	JAWS II	4.8	*5*	(35)
rAmb a 1.0301	Short ragweed	*Ambrosia artemisiifolia*	Pollen	Inhalant	Pectate lyase	40	5.4	A20	4.8	*42*	(35)
rAmb a 1.0301	Short ragweed	*Ambrosia artemisiifolia*	Pollen	Inhalant	Pectate lyase	40	5.4	RAW 264.7	4.8	*>72*	(35)
rAmb a 1.0301	Short ragweed	*Ambrosia artemisiifolia*	Pollen	Inhalant	Pectate lyase	40	5.4	Activated RAW 264.7	4.8	*>72*	(35)
nAmb a 1.01	Short ragweed	*Ambrosia artemisiifolia*	Pollen	Inhalant	Pectate lyase	40	5.3	JAWS II	4.8	45	(36)
nAmb a 1.01	Short ragweed	*Ambrosia artemisiifolia*	Pollen	Inhalant	Pectate lyase	40	5.3	JAWS II	4.5	12	(36)
rArt v 1.0101	Mugwort	*Artemisia vulgaris*	Pollen	Inhalant	Defensin-polyproline-linked protein	11	8.2	Human mDCs	4.8	*48–72*	(45)
rAmb a 4.0101	Short ragweed	*Ambrosia artemisiifolia*	Pollen	Inhalant	Defensin-polyproline-linked protein	14	4.9	Human mDCs	4.8	*0.5–1*	(45)
rPar h 1.0101	Santa Maria feverfew	*Parthenium hysterophorus*	Pollen	Inhalant	Defensin-polyproline-linked protein	12	5.3	Human mDCs	4.8	*1–3*	(45)
nPru p 3	Peach	*Prunus persica*	Fruit	Ingested	nsLTP type 1	9	9.3	Mouse BMDCs	4.8	*30*	(39)
nPru p 3 (reduced/alkylated)	Peach	*Prunus persica*	Engineered	–	nsLTP type 1	9	9.3	Mouse BMDCs	4.8	*1*	(39)
rPru p 3.0102	Peach	*Prunus persica*	Fruit	Ingested	nsLTP type 1	9	9.3	Human mDCs	4.8	32	(46)
nPru 3.0102	Peach	*Prunus persica*	Fruit	Ingested	nsLTP type 1	9	9.3	Human mDCs	4.8	*27*	(47)
rCor a 8.0101	Hazelnut	*Corylus avellana*	Tree nut	Ingested	nsLTP type 1	10	9.3	Human mDCs	4.8	*12*	(47)
rArt v 3.0201	Mugwort	*Artemisia vulgaris*	Pollen	Inhalant	nsLTP type 1	10	8.8	Human mDCs	4.8	11	(46)
rApi g 2.0101	Celery stalk	*Apium graveolens*	Vegetable	Ingested	nsLTP type 1	9	9.4	Human mDCs	4.8	18	(46)
rApi g 6.0101	Celery tuber	*Apium graveolens*	Vegetable	Ingested	nsLTP type 2	7	9.2	Human mDCs	4.8	17	(48)
rPro-Der p 1.0102	European house dust mite	*Dermatophagoides pteronyssinus*	Fecal pellets	Inhalant	Mite group 1, cysteine protease	34	5.5	JAWS II	4.8	*42*	(35)
rPro-Der p 1.0102	European house dust mite	*Dermatophagoides pteronyssinus*	Fecal pellets	Inhalant	Mite group 1, cysteine protease	34	5.5	A20	4.8	*>72*	(35)
rPro-Der p 1.0102	European house dust mite	*Dermatophagoides pteronyssinus*	Fecal pellets	Inhalant	Mite group 1, cysteine protease	34	5.5	RAW 264.7	4.8	*24*	(35)
rPro-Der p 1.0102	European house dust mite	*Dermatophagoides pteronyssinus*	Fecal pellets	Inhalant	Mite group 1, cysteine protease	34	5.5	Activated RAW 264.7	4.8	*12*	(35)
rDer p 2.0103	European house dust mite	*Dermatophagoides pteronyssinus*	Fecal pellets	Inhalant	Mite group 2 allergen, MD-2 related	14	7.1	JAWS II	4.8	*>72*	(35)
rDer p 2.0103	European house dust mite	*Dermatophagoides pteronyssinus*	Fecal pellets	Inhalant	Mite group 2 allergen, MD-2 related	14	7.1	A20	4.8	*>72*	(35)
rDer p 2.0103	European house dust mite	*Dermatophagoides pteronyssinus*	Fecal pellets	Inhalant	Mite group 2 allergen, MD-2 related	14	7.1	RAW 264.7	4.8	*>72*	(35)
rDer p 2.0103	European house dust mite	*Dermatophagoides pteronyssinus*	Fecal pellets	Inhalant	Mite group 2 allergen, MD-2 related	14	7.1	Activated RAW 264.7	4.8	*>72*	(35)
rBlo t 5.0101	Tropical mite	* Blomia tropicalis*	Fecal pellets	Inhalant	Mite group 5 allergen	14	5.3	JAWS II	4.8	*4*	(49)
rBlo t 5.0101 short, N-terminal motif removed	Tropical mite	* Blomia tropicalis*	Fecal pellets	Inhalant	Mite group 5 allergen	12	5.2	JAWS II	4.8	*11*	(49)
rBlo t 5.0101 short, N-terminal motif removed	Tropical mite	* Blomia tropicalis*	Fecal pellets	Inhalant	Mite group 5 allergen	12	5.2	JAWS II	4.8	9	(38)
rBlo t 21.0101 short, N-terminal motif removed	Tropical mite	* Blomia tropicalis*	Fecal pellets	Inhalant	Mite group 21 allergen	11	5.4	JAWS II	4.8	17	(38)
rBTH1 (Blo t 5/21 hybrid WT sequence, N-terminal motif removed)	Tropical mite	* Blomia tropicalis*	Engineered	–	Hybrid mite group 5/21 allergen	11	4.9	JAWS II	4.8	39	(38)
rBTH2 (Blo t 5/21 hybrid, stability variant, N-terminal motif removed)	Tropical mite	* Blomia tropicalis*	Engineered	–	Hybrid mite group 5/21 allergen	11	4.8	JAWS II	4.8	9	(38)
rPen m 1.0101	Black tiger shrimp	*Penaeus monodon*	Muscle tissue	Ingested	Tropomyosin	33	4.7	JAWS II	5.2	*24–48*	(37)
rPen m 1.0101	Black tiger shrimp	*Penaeus monodon*	Muscle tissue	Ingested	Tropomyosin	33	4.7	JAWS II	4.5	*24–48*	(37)
rDer p 10.0101	European house dust mite	*Dermatophagoides pteronyssinus*	Fecal pellets	Inhalant	Tropomyosin	33	4.8	JAWS II	5.2	*12–24*	(37)
rDer p 10.0101	European house dust mite	*Dermatophagoides pteronyssinus*	Fecal pellets	Inhalant	Tropomyosin	33	4.8	JAWS II	4.5	*24–48*	(37)
rBla g 7.0101	German cockroach	*Blattella germanica*	Fecal pellets	Inhalant	Tropomyosin	33	4.7	JAWS II	5.2	*12–24*	(37)
rBla g 7.0101	German cockroach	*Blattella germanica*	Fecal pellets	Inhalant	Tropomyosin	33	4.7	JAWS II	4.5	*12–24*	(37)
rAni s 3.0101	Herring worm larvae	* Anisakis simplex*	Fish muscle tissue	Ingested	Tropomyosin	33	4.7	JAWS II	5.2	*8–12*	(37)
rAni s 3.0101	Herring worm larvae	* Anisakis simplex*	Fish muscle tissue	Ingested	Tropomyosin	33	4.7	JAWS II	4.5	*12–24*	(37)

Allergen designations according to WHO/IUIS Allergen Nomenclature Sub-committee. Half-lives in italic represent estimated half-lives obtained with densitometric analyses using protein gel electrophoresis data provided in the original publications. Recombinant allergens were obtained from *E. coli*, except for Ole e 1, Fra e 1, Sal k 5, Che a 1 and Phl p 11 which were produced in *P. pastoris*.

r, recombinant; n, natural; nsLTP, non-specific lipid transfer protein; PR, pathogenesis-related.

The activity of endolysosomal degradation is regulated according to the biological function of the APCs. Compared to DCs and B cells, macrophages present higher levels of proteases, resulting in enhanced degradation of casein and ovalbumin (5). Specifically, the activity of cysteine proteases determined by a peptide-based assay in murine cell lines was shown to be 6.3-fold higher in endolysosomes of RAW 264.7 macrophages but 151-fold lower in A20 B cells compared to JAWS II (35). Thus, the amount of endolysosomal proteins in the degradation assay of this study was adjusted, but not fully equivalent, as shown by the different degradation kinetics of Bet v 1 (Table 1). Standardization on the activity of the entire protease panel is required for efficient comparison of endolysosomal proteases from different antigen-presenting cells and studies performed in different laboratories. Among endolysosomal proteases, cathepsins are the most abundant protease family (51). For example, digestion of Bet v 1 with purified cathepsin S generated 8 out of 13 endolysosomal peptide clusters, demonstrating the involvement of cathepsin S in early protein processing (6). Ole e 1, the major olive pollen allergen is typically completely digested within 8 h, while this process was considerably delayed to more than 36 h when a cathepsin S inhibitor was used. Cathepsin S-inhibited degradation showed similar peptide clusters, albeit with limited peptide diversity and up to 28-fold reduction in the total peptide amounts (34).

### Proteolytic stability, endolysosomal peptide clusters and antigenicity of allergenic proteins

**Figure 2:**
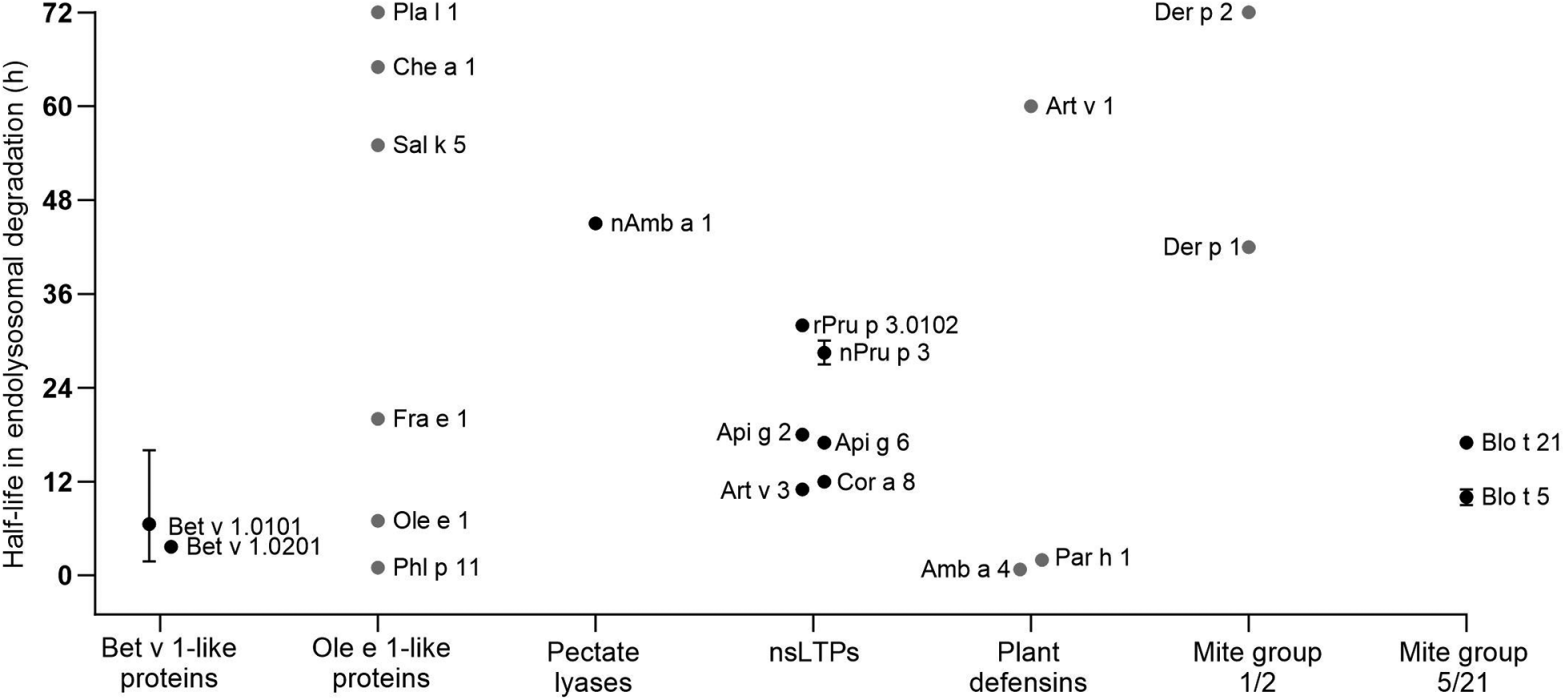
Half-lives of allergens observed by endolysosomal degradation (for comparison only values obtained for digestion at pH 4.8 and proteases from DCs are given). Allergen isoforms correspond to those given in Table 1; values for Blo t 5 correspond to Blo t 5 short. Black dots represent values provided in the reviewed papers. Grey dots represent estimated half-lives determined by densitometric analyses of protein gel data provided in the original papers. Whiskers indicate highest and lowest half-lives from different endolysosomal degradations, dots represent the mean values.

In this chapter, detailed information on the endolysosomal stability of 29 allergens from ten protein families obtained from the literature is provided (Table 1). The stability of each protein is expressed as half-life, defined as timepoint when 50% is digested. If the half-life was not provided in the studies, we determined the values by densitometric analyses using protein gel electrophoresis data retrieved from the original papers. Purified allergens were obtained from natural sources or produced in *E. coli*, except for Ole e 1, Fra e 1, Sal k 5, Che a 1 and Phl p 11 which were produced in *P. pastoris*. These allergens were digested with proteases obtained from the endolysosomal compartments of mainly DCs and in some cases from B cells and macrophages. No obvious association between endolysosomal half-life and allergenic sensitization capacity is detectable and major differences within protein families are noted (Figure 2). However, allergens of minor sensitization prevalence typically showed very low half-lives, while the majority with medium half-lives are considered moderate to strong allergenic sensitizers. While the endolysosomal stability is generally relevant for immunogenicity, further intrinsic or context-related factors seem to further influence the development of a Th2 response and allergy.

From the literature, we additionally collected information on peptide clusters identified by mass spectrometry. These clusters are typically longer than the presented *T* cell epitopes, as peptides are additionally trimmed. However, these clusters provide an important overview of the relevant peptides and the kinetics of their appearance. To allow comparison between different studies, the numbering of *T* cell epitopes and identified clusters is provided considering the mature proteins without signal peptides, unless otherwise stated. Finally, the antigenic potential of the investigated proteins is discussed when immunization data were available.

#### Bet v 1-like, pathogenesis-related 10 proteins

Bet v 1 is the major allergen of birch pollen with a sensitization frequency of 95% and belongs to the plant pathogenesis related-10 (PR-10) protein family (52). Twenty-seven isoallergens have been identified to date, with Bet v 1 isoforms and fold variants being the most studied molecules with respect to endolysosomal degradation (32, 53). Studies with endolysosomal proteases from DCs showed half-lives of Bet v 1.0101 ranging from 1.8 to 14 h (6, 35, 41). The first studies showed comparatively shorter half-lives of 2 and 5 h (6, 27), while further work typically suggested longer periods of time. The observed discrepancies for the half-lives of Bet v 1.0101 might relate to different protein quantification methods used for determining protein concentrations of the isolated protein as well as for the endolysosomal fraction and/or differences in protease activity in the endolysosomal protein cocktail. During simulated endolysosomal degradation of allergens, the degradation buffer of the assay is typically maintained at pH 4.8 (6, 27). To specifically address the pH difference in the antigen processing compartments, a study on kinetics and peptide clusters of Bet v 1 was performed with pH conditions of 5.9, 5.2, and 4.5, simulating early to late endosomal compartments (33). Degradation of Bet v 1 with JAWS II endolysosomal proteases demonstrated that the allergen was degraded significantly faster at lower pH, while at pH 5.9 intact protein was still present after 48 h. Furthermore, Bet v 1 was completely degraded at pH 5.2 after 24 h and at pH 4.5 already after 6 h (33).

Recombinant Bet v 1.0101 and *E. coli* DNA codon-harmonized Bet v 1.0101 were compared regarding structural and immunological aspects (40). Regarding endolysosomal degradation, similar half-lives of 7 and 8 h were observed for Bet v 1.0101 and the codon-harmonized protein, respectively. Both proteins also showed comparable IgE binding capacities suggesting equivalent 3-dimensional structures. To alter the immunomodulatory properties of Bet v 1 by ligand binding, retinoic acid bound Bet v 1.0101 was investigated. Using JAWS II cells, the endolysosomal degradation kinetics of Bet v 1.0101 remained the same when retinoic acid was bound to the allergen, although allergenicity was shown to be reduced (42). On the other hand, coupling of Bet v 1.0101 to SiO_2_ nanoparticles resulted in significantly lower stability during endolysosomal degradation with JAWS II proteases (43). The estimated half-life was strongly reduced from 14 h to only 1 h. The difference suggests that conjugation with SiO_2_ nanoparticles resulted in conformational changes of Bet v 1.0101 leading to increased exposure of proteolytic cleavage sites. At the same time, IgE and *T* cell reactivity were similar but SiO_2_ conjugated Bet v 1.010 showed a more pronounced Th1 profile (54).

Isoform Bet v 1.0201 (previously known as Bet v 1.0401 or Bet v 1d) is considered a naturally occurring hypoallergen based on lower IgE binding and retained *T* cell activation compared to Bet v 1.0101 (6, 55). Endolysosomal degradation with proteases isolated from mouse BMDC, human mDC and JAWS II showed that Bet v 1.0201 is more resistant to proteolysis (half-lives 3.9–4.1 h) compared to Bet v 1.0101 (half-lives 1.8–2.1 h) (6). In another study, Bet v 1.0201 also showed slightly increased stability to endolysosomal degradation compared to Bet v 1.0101 (42). Mice immunized with Bet v 1.0201 showed similar IgE responses, whereas higher IgG and IgA levels were observed consistent with enhanced antigen uptake of isoform 1.0201 (56). Based on *in silico* calculations, local unfolding of Bet v 1.0201 provides more accessible cleavage sites, which would argue for increased proteolytic susceptibility (57, 58). In contrast to Bet v 1.0101, Bet v 1.0201 exists as cysteine-mediated dimer which might explain the difference between theoretical and observed accessibility to proteolysis.

An engineered fold variant of Bet v 1 (BM4) incorporating 7 consecutive amino acids from the homologous apple allergen Mal d 1 showed a loss of the typical PR-10 fold (41). Endolysosomal degradation of BM4 with mouse BMDC-derived proteases resulted in a half-life of 9 h (complete degradation at 24 h) compared to 6 h (complete degradation after 12 h) for Bet v 1.0101. In another study, BM4 proved to be more susceptible to endolysosomal degradation with a half-life of approximately 0.5 compared to 2 h for Bet v 1.0101 (44). In addition, BM4 was taken up more efficiently by human PBMCs and thus faster intracellular degradation was observed supporting the lower resistance to endolysosomal degradation (44). This observed discrepancy in stability might be attributed to the fact that proteases in the two different studies were obtained from mouse BMCDs and human mDCs. In addition, variations in batches and determination of protein concentrations would influence proteolysis, which is however beyond identifiable data.

Immunogenicity testing of BM4 with PBMCs from birch pollen allergic patients and Bet v 1-specific *T* cell clones showed higher *T* cell proliferation compared to Bet v 1.0101 (41, 44). Immunization of mice with BM4 triggered higher levels of Bet v 1-specific IgG_1_ and IgG_2a_ and IgE compared to Bet v 1.0101. While cytokine profiles indicated a Th2 polarization using Bet v 1.0101, immunization with BM4 resulted in a mixed Th1/Th2 response. As mentioned above, two different patterns were observed showing both higher and lower susceptibility of BM4 compared to Bet v 1.0101 to endolysosomal degradation. It was suggested that higher endolysosomal stability of BM4 led to the observed shift towards a Th1 immune response. On the other hand, higher susceptibility was attributed to increased access to proteolytic sites in the fold variant BM4 (41, 44).

**Figure 3:**
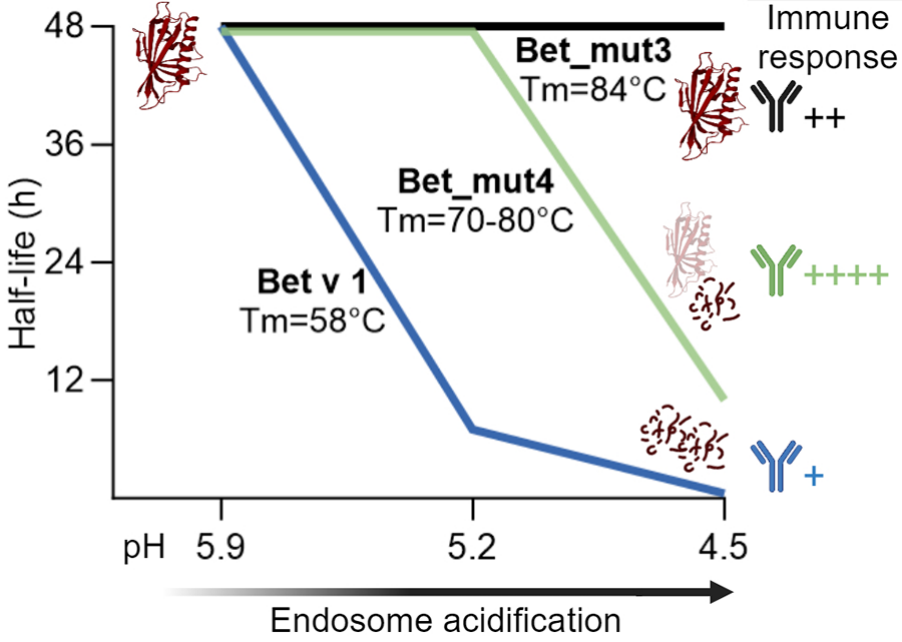
Schematic representation of endolysosomal stability depicted as half-life for Bet v 1, stabilized variant Bet_mut4 and hyperstabilized variant Bet_mut3 at different pH conditions. Bet v 1 and variants showed different antibody responses in adjuvant free mouse immunizations performed by Machado et al. (33).

Proteolytic cleavage sites of Bet v 1 are mostly located within inaccessible secondary structural elements, thus local unfolding is required to expose proteolytic sites. For that reason, structural stability and unfolding ability are thought to be intrinsically linked to protein degradation (58). To test this hypothesis, four fold-stabilized variants of Bet v 1.0101 designated Bet_mut1 (D69I), Bet_mut2 (D69I, K97I), Bet_mut3 (D69I, K97I, P90l), Bet_mut4 (D69I, K97I, P90l, G26l), were generated with similar three-dimensional structure but strongly enhanced thermal and chemical stability (59). In the endolysosomal degradation assay, wild-type Bet v 1 and variants were digested at different acidic conditions (pH 5.9, 5.2, and 4.5) (Figure 3). All proteins remained intact when digested at pH 5.9, which mimics the early endosomes milieu. Bet v 1.0101 showed higher susceptibility to proteolysis at pH 5.2 (half-life 7 h) and pH 4.5 (half-life 0.5 h) compared to all fold-stabilized variants which (expect for Bet_mut4) showed significantly higher endolysosomal stability (Table 1). Bet_mut4 was only efficiently degraded at pH 4.5 (half-life 10 h). The endolysosomal degradation results are consistent with the thermal and chemical stability of the molecules.

The immunogenicity of the proteins was tested by intradermal administration in BALB/c mice (33). While Bet v 1.0101 did not induce an immune response without adjuvant, Bet_mut4 showed the highest immunogenicity and allergenicity as evidenced by strong IgG, IgE and IL-4 responses. Bet_mut1, which is most similar to Bet v 1.0101, showed only very weak responses, while Bet_mut2 and Bet_mut3 showed intermediate responses. Interestingly, no differences were observed in antigen uptake experiments, suggesting that antigen processing is most relevant for the observed immunological differences *in vivo*. A Jurkat *T* cell line specific for the immunodominant Bet v 1_142−153_ epitope showed enhanced activation by Bet_mut4 and to a lesser extent Bet_mut3. This study demonstrates that changes of only a few amino acids can lead to increased endolysosomal stability, resulting in altered immunogenicity. In summary, studies on Bet v 1 and variants demonstrate that stabilization of proteins typically leads to enhanced peptide presentation and thus higher immunogenicity of the protein. It seems particularly relevant that proteins are stable in the pH of the early endosome milieu but are later efficiently digested at lower pH as exemplified by Bet_mut4 (Figure 3). Proteins that are too susceptible (e.g., Bet v 1.0101) or on the other hand too stable (e.g., Bet_mut3) result in low peptide abundance and insufficient MHC loading in the late endosomes as previously suggested (41, 44).

Peptide clusters generated by endolysosomal degradation of Bet v 1.0101 are typically located around residues 1-22, 83-115 and, to a lesser extent around 146-159. They all contain the previously identified *T* cell epitopes, i.e., Bet v 1_4−18_, Bet v 1_82−96_, and Bet v 1_142−156_. In addition to the regions containing *T* cell epitopes, there was also a strong peptide cluster located at 36-55 (6, 25, 27, 35, 41, 43, 60, 61). To investigate whether peptides generated by endolysosomal degradation are presented on MHC II molecules, Mutschlechner et al. eluted peptides from HLA-DR molecules obtained from Bet v 1-allergic donors and identified them using mass spectrometry (33). This method is able to accurately identify *T* cell epitopes but is more demanding than mapping with synthetic peptides. The eluted N-terminal peptides were located between residues 4–32 (three of four patients) and 69–96 (one patient). All found regions were previously identified as *T* cell epitopes and were also present in the endolysosomal degradation clusters. In addition, for 2 patients a peptide was found originating from the C-terminus, i.e., 132–159, which encompasses the major Bet v 1 *T* cell epitope. In endolysosomal degradation, the peptide cluster at 146–159 partially overlaps with this relevant region (6). However, this cluster contained only a few different peptides and was not among the most prominent peptides found upon endolysosomal degradation. Mutschlechner et al. suggested that this region might be proteolytically protected *in vivo* by binding to MHC II, while being degraded and thus undetectable in the endolysosomal degradation assay (27). On the other hand, several degradation studies showed that some peptides containing the major *T* cell epitope were identified but possibly underestimated as quantification of peptides was not provided. In conclusion, it is possible to use the endolysosomal degradation assay to identify experimental *T* cell epitopes and most importantly *T* cell peptides presented on HLA molecules of patients.

Endolysosomal degradation of the Bet v 1.0201 isoform with DC proteases produced peptide clusters similar to those of Bet v 1.0101. However, in accordance with the higher endolysosomal stability, cluster formation was slightly delayed (6, 25, 27, 35, 41, 43, 60, 61). In general, the peptide cluster containing the *T* cell epitope Bet v 1_82−96_ showed lower number of peptides, especially for JAWS II-derived proteases. Several studies on modified Bet v 1.0101 have focused on endolysosomal clustering. Similar peptide cluster profiles were observed for Bet v 1.0101 coupled to SiO2 nanoparticles as well as the BM4 fold variant (33). An engineered hybrid termed MBC4, consisting of Bet v 1.0101, Mal d 1.0108 and Cor a 1.0401 showed similar but earlier cluster formation after 1 h of digestion compared to Bet v 1.0101 (25). Endolysosomal peptide pattern of Bet v 1 and fold-variants were analyzed additionally for digestions at pH 5. 2 and pH 4.5. At both pH conditions, similar peptide clusters were observed for Bet v 1 as well as the variants. Those clusters also corresponded to previously identified clusters when digestion was performed at pH 4.8. However, the kinetics of peptide formation was substantially influenced by the different buffers, indicating that Bet v 1 is most prone to endolysosomal proteolysis when performed at pH 4.5 (27).

Information on endolysosomal degradation of other Bet v 1-like allergens from food sources was also found. Variants of the major allergenic apple and hazelnut PR-10 proteins Mal d 1.0108 and Cor a 1.0401 were generated to compare immunogenicity, allergenicity and proteolytic susceptibility with the respective wild-type allergens (60). Fold variants aimed to destabilize the proteins while cysteine variants should prevent disulfide-bond induced oligomerization. All variants showed reduced human IgE binding capacities, lower IgG1 immune responses in mice and higher proteolytic susceptibility to JAWS II endolysosomal proteases compared to wild-type allergens (60). Further details on endolysosomal degradation kinetics were not provided.

Endolysosomal peptide clusters of Mal d 1.0108 and Api g 1.0101 obtained by degradation with moDCs were located within residues 1–22, 23–36, 83–102, 146–157 (early appearing peptides) followed by 33–55, 56–65, and 103–115 (late appearing peptides). The same localization of peptides clusters were previously also identified in Bet v 1.0101 digestions (62). Similar peptide cluster formation was also observed for fold and cysteine variants of Mal d 1.0108 and Cor a 1.0401, analogous to Bet v 1 (60). The formation of such highly similar peptide clusters is interesting, because the allergens studied have sequence identities of 42%–67% to Bet v 1. This indicates that in addition to sequence identity, the tertiary structure and thus accessibility of protease cleavage sites is important for peptide generation. Apart from the observed similar clusters, it is relevant to note that PR-10 food allergens (class 2) usually do not act as primary sensitizer, but mostly cause IgE-related symptoms due to Bet v 1 induced cross-reactive antibodies (33). The exposure route of PR-10 food allergens and a potential primary sensitization capacity would additionally require simulation of a gastrointestinal digestion before the endolysosomal degradation assay (62).

#### Ole e 1—like proteins

The Ole e 1-like protein family includes 15 pollen-derived allergens, the biological function of which is still unknown. The prototypic member Ole e 1 is the major allergen of olive pollen which causes seasonal allergic reactions predominately in the Mediterranean area (63). Other allergenic family members are Fra e 1 from ash, Sal k 5 from Russian thistle, Che a 1 from chenopod, Phl p 11 from grass and Pla l 1 from English plantain pollen. Although the three-dimensional structure of these proteins is highly similar, they show a wide range of sequence identity ranging from 25% to 89% (34). Endolysosomal degradation studies with six allergenic Ole e 1-like allergens revealed different susceptibility to endolysosomal proteases isolated from JAWS II. Sal k 5 and Che a 1 share 72% sequence identity (identity to Ole e 1 is only 36%) and their half-lives during endolysosomal degradation were similar with ∼55 and ∼65 h, respectively. Despite 89% sequence identity, Ole e 1 and Fra e 1 showed half-lives of ∼7 and ∼20 h, respectively. The higher resistance to degradation of Fra e 1 could possibly be explained by the yeast-specific glycosylation of recombinant Fra e 1 (64). Phl p 11, a minor allergen from timothy grass, has a half-life of only ∼1 h, while the major English plantain allergen Pla l 1 was very resistant to endolysosomal degradation for up to 72 h. Ole e 1 and Fra e 1 are potent allergens which showed moderate stability during endolysosomal degradation. Sal k 5, Che a 1 and Pla l 1 also produced sufficient peptides over a longer period. On the other hand, Phl p 11 seems to be digested too fast for efficient and stable presentation on MHC II, which explains its low relevance as a grass pollen allergen.

*T* cell epitope mapping studies with Ole e 1 revealed immunodominant epitopes at residues 91–102, 109–120, and 119–130 (65). Notably, most of the relevant peptide clusters from endolysosomal degradation (83–104, 105–121, and 122–139) correspond with the experimentally verified *T* cell epitopes (34). On the other hand, the *in silico* predicted epitopes in the same study showed high genetic heterogeneity due to HLA restricted predictions and only partially overlapped with the experimentally found *T* cell epitopes. The findings indicate that *in silico* *T* cell epitope prediction is not yet optimal and more robust peptide data for Ole e 1 were obtained using the endolysosomal degradation assay.

Three *in silico* predicted Fra e 1 peptides were tested using human PBMCs and confirmed 21–35, 35–45 and 121–135 as *T* cell epitopes (66). Endolysosomal degradation of Fra e 1 yielded a cluster at 120–138 within and 27–41 partially overlapping with the identified *T* cell epitopes (34). Interestingly, i*n silico* predicted epitopes were suggested at positions where strong protease cleavage was observed in the endolysosomal degradation assay. On the other hand, the endolysosomal degradation assay revealed a dominant peptide cluster between 83 and 105, not found with *in silico* prediction, with strong proteolytic sites located within predicted *T* cell epitopes. The predicted peptides could potentially be biased due to HLA binding restrictions, and additional epitopes may exist that could only be revealed when testing peptides covering the entire protein sequence.

Phl p 11 presents one experimentally verified *T* cell epitope at residues 111–125. However, the number of peptides tested was small and ultimately validated in only one patient (67). Although few peptides from the endolysosomal degradation assay contain the proposed *T* cell peptide, most peptides within this region are enzymatically cleaved in the middle of the proposed epitope. Several other potentially relevant regions were identified in the endolysosomal degradation assay that partially overlap with the *in silico* predicted *T* cell epitopes. Pla l 1 epitopes 21–35 and 76–90 were identified in the same study (67), both of which are included in peptide clusters from the endolysosomal degradation (34). In addition, a dominant C-terminal peptide cluster was found at 94-122, which overlaps with a predicted *T* cell epitope. Furthermore, the peptides do not form strong clusters, but an *in silico* epitope at 62–71 was identified at longer digestion times. To unravel the *T* cell epitopes of Phl p 11 and Pla l 1, it would be necessary to test peptides covering the entire sequence and to include more patients.

For Sal k 5 and Che a 1, data are limited to *in silico* prediction of *T* cell epitopes. Both allergens show several peptide clusters containing partially predicted epitopes after endolysosomal degradation. However, some *in silico* predicted epitopes included dominant proteolytic cleavage sites in the degradation assay. Using the endolysosomal degradation assay, six allergenic Ole e 1-like proteins were analyzed (34). Aside from Ole e 1, there is a general lack of systematic *T* cell epitope mapping that considers the entire sequence for peptide mapping and therefore more research is needed on this allergen family.

#### Pectate lyases

Amb a 1, a member of the pectate lyase C family, is the major allergen of short ragweed, accounting for 90% of sensitization and 50% of IgE reactivity in ragweed pollen (68, 69). Interestingly, endolysosomal degradation with JAWS II proteases at pH 4.8 revealed different half-lives of 5 h for recombinant Amb a 1.0301 and 45 h for natural Amb a 1.01 (35, 36). The observed differences could be related to the Amb a 1 isoform and/or to the protein source, which was recombinantly expressed in bacteria or purified from pollen. Digestion of nAmb a 1.01 at pH 4.5 showed a reduced half-life of 12 h (36). In comparison, a non-allergenic bacterial pectate lyase was not degraded even after 72 h, regardless of the buffer conditions used. As expected, fewer peptides were generated by the bacterial pectate lyase and differences in peptide clusters are explained by the low sequence similarity (36). To investigate the immunogenicity, mouse studies were performed with subcutaneously administered nAmb a 1.01 and the bacterial homolog. nAmb a 1.01 induced a strong IgG and IgE response after immunization. The bacterial homolog showed reduced immunogenicity, and IgG levels were particularly lower after immunization without the aluminum hydroxide adjuvant. Compared to nAmb a 1.01 with medium stability and high immunogenicity, the extreme endolysosomal stability of the bacterial pectate lyase resulted in lower immunogenicity and additionally a shift towards a Th1 immune response (36).

Amb a 1 epitope mapping studies revealed dominant *T* cell epitopes at Amb a 1 residues 178–189, 199–216, 280–295, 304–319, 320–335, 343–357, and 361–394 (70, 71). Endolysosomal degradation of rAmb a 1 with the DC line JAWS II, the macrophage cell line RAW 264.7, and the B cell line A20 proteases resulted in peptide clusters at residues 121–149, 172–191, 283–296, 337–353, and 356–383, which incorporate four of the above mentioned *T* cell epitopes (35, 70, 72). Peptide clusters at residues 203–211 and 227–243 were only observed using JAWS II proteases and overlap with the *T* cell epitope 199–216. In addition, peptide clusters around residues 89–108, 264–277 and 310–326 of Amb a 1 were observed using endolysosomal degradation, whereas these regions do not contain previously identified *T* cell epitopes. On the other hand, epitopes 304–319 and 320–335 were not represented in endolysosomal peptide clusters (35). The endolysosomal proteases from the different antigen-presenting cells generally showed very similar peptide clusters, although the kinetics were different. In the first 6 h, JAWS II proteases produced the most peptides, but at the end of 24 h, A20 proteases produced the most peptides (35). In another study, endolysosomal degradation of natural Amb a 1.01 by JAWS II proteases for 48 h resulted in the formation of peptide clusters that covered almost the entire protein sequence, and included identified *T* cell epitopes (36).

#### Non-specific lipid transfer proteins

Non-specific lipid transfer proteins (nsLTPs) are relevant primary food allergens in southern Europe and parts of Asia (73). They are widespread in the plant kingdom and their physiological role includes plant defense against bacteria and fungi (pathogenesis-related protein 14). Their cysteine-stabilized alpha-helical fold results in high thermal and proteolytic stability which may be responsible for severe allergic reactions. Due to their conserved structure, frequent antibody cross-reactivity is observed between botanically unrelated members (74–77). There are two types, nsLTP1 (9 kDa) and nsLTP2 (6 kDa), with more allergens and higher allergenic potential found in type 1 (78, 79).

Pru p 3 is the major peach allergen and the prototypical allergen of the nsLTP1 family (80). Pru p 3 is highly thermostable, especially under acidic conditions, and shows high stability in simulated gastrointestinal digestions (39, 81). Degradation of Pru p 3 with endolysosomal proteases revealed half-lives of 27 and 32 h (mDCs from nsLTP-allergic donors) and 30 h (mouse BMDCs) (39, 46). In contrast, a reduced/alkylated hypoallergenic Pru p 3 variant was degraded by endolysosomal proteases of mouse BMDCs already after 1 h, due to protein unfolding. While B cell reactivity of this variant was diminished, *T* cell reactivity was interestingly still preserved (39). Immunodominant *T* cell epitopes were identified as Pru p 3 residues 13–27, 25–35, 34–48, 43–57, 61–75 (39, 82). All epitopes were located within the endolysosomal peptide clusters (residues 10–36, 30–47, 38–52, 54–74). In addition, a C-terminal cluster was found at residues 77–91 (39, 46, 47). The reduced/alkylated Pru p 3 variant showed similar peptide clusters as wild-type Pru p 3 but appeared already at earlier time points due to rapid degradation (39).

Celery can cause allergic reactions ranging from mild oral allergy syndromes to severe anaphylactic reactions associated with highly stable allergens (83). Api g 2.0101 belongs to the nsLTP1 type and is found in celery stalks. Similar to other nsLTPs, it is highly resistant to thermal and proteolytic treatment, especially in an acidic environment (84). Endolysosomal degradation with mDC from nsLTP-allergic donors showed a half-life of 18 h (46). Api g 6.0101, is an allergen found only in celery tuber and belongs to the nsLTP2 type. Api g 6 is highly heat resistant and also extremely stable during gastrointestinal digestion, which has been attributed to the highly compact folding of nsLTP2 (48). Endolysosomal degradation of Api g 6 with mDC of LTP-allergic donors showed a half-life of 17 h. Endolysosomal degradation of Api g 2.0101 with proteases from nsLTP-allergic patients revealed peptide clusters at residues 6–33, 29–47, 54–74 and 75–91. For Api g 6.0101, a dominant peptide cluster was observed at residues 4–23, 13–35 and 38–58. No *T* cell epitope mapping studies of Api g 2 or Api g 6 are available. However, the Api g 2 peptide clusters include *T* cell epitope regions previously identified for Pru p 3 (39, 82).

Art v 3 is a relevant allergenic nsLTP1 from mugwort pollen. Endolysosomal degradation with mDC of nsLTP allergic donors showed a half-life of 11 h (46). Art v 3 is highly thermostable (T_m_ 90°C), but in contrast to other nsLTPs it is degraded within 10 min during simulated gastric digestion (81, 85). Endolysosomal degradation of Art v 3 with proteases of nsLTP allergic donors revealed peptide clusters at 5–33, 29–47, 52–71 and 75–91 (46). Regarding immunogenicity, higher IgG antibody responses in mice were observed for Art v 3 compared to Pru p 3 and Api g 2 (46). This may be related to the shorter half-life of Art v 3 and thus earlier accessibility of the peptides required for antigen presentation. Cor a 8, the nsLTP1 from hazelnut is completely digested within 24 h using endolysosomal proteases from mDC-allergic donors (estimated half-life 12 h). Three peptide clusters were identified at residues 33–57, 58–69 and 72–91 (47). Interestingly, no immunodominant *T* cell epitope was identified in peptide mapping experiments, but some reactivity was observed with Cor a 8 peptides within residues 46–75.

#### Plant defensins

Defensins are ubiquitous plant peptides that play a role in pathogen defense (PR-12 family). They possess a disulfide bond stabilized structure that provides high resistance to extreme pH, temperature and protease degradation. Allergenic defensins from pollen additionally contain a C-terminally linked polyproline domain designated defensin polyproline-linked protein (DPLP). Particularly Art v 1 from mugwort pollen shows cross-reactivity with food defensins, which can lead to pollen food syndromes (7, 86). Allergic reactions to mugwort pollen are observed in Asia, Europe, and North America. The major allergen, Art v 1, is a DPLP recognized by IgE of 95% of mugwort pollen allergic patients (87). Digestion of Art v 1 with endolysosomal proteases of the mouse cell line JAWS II showed that the defensin domain was stable for 72 h, while the polyproline region was digested within 3 h (45). Ragweed pollen allergy is prevalent in North America, Europe, and parts of Asia and Amb 4 accounts for 20%–40% of IgE reactivity in ragweed sensitized patients (7, 88). Amb a 4-defensin domain was stable to degradation by mouse DC line-derived endolysosomal proteases for up to 8 h, while the polyproline region was intact for up to 72 h (45). Feverfew is an allergenic weed that causes allergic reactions including contact dermatitis in the USA and India (7, 89). The DPLP allergen Par h 1 is IgE reactive with sera from weed allergic patients from Canada, Korea and Austria, but this is mainly due to IgE cross-reactivity (90). The Par h 1-defensin domain was stable up to 16 h of endolysosomal digestion using a mouse DC line, while the polyproline region was intact up to 72 h (45). The fact that the Art v 1-defensin domain, which contains all relevant *T* cell epitopes, shows significantly higher endolysosomal stability than Amb a 4 and Par h 1 defensin domain is consistent with the higher primary sensitizing capacity and allergenic relevance of Art v 1 (45).

Endolysosomal degradation of Art v 1, Amb a 4 and Par h 1 revealed similar peptide clusters within the analyzed defensin-like domain. For all three allergens, a large peptide cluster spanning residues 25–43 was observed (45). Notably, this region includes the immunodominant *T* cell epitope Art v 1_25−36_, suggesting that the identified endolysosomal peptides are trimmed from both ends to the central core of the *T* cell epitope (45, 87, 91). In a *T* cell line highly specific for the immunodominant Art v 1_25−36_ peptide, reactivity was observed for Art v 1, but not for Amb a 4 or Par h 1, suggesting a limited *T* cell cross-reactivity within this peptide region under the highly specific assay settings (45). The *T* cell epitopes of Amb a 4 and Par h 1 remain to be determined experimentally. Another peptide cluster was found at Art v 1_8−21_, which was however not identified as a *T* cell epitope (26).

#### Mite group 1 and 2 allergens

Der p 1 and Der p 2 are two major house dust mite allergens found in the fecal pellets and account for >80% of IgE reactivity in house dust mite allergic individuals (92). Endolysosomal degradation of recombinant pro-Der p 1 (Der p 1.0102 including the N-terminal pro-peptide) with proteases from JAWS II and A20 revealed high proteolytic resistance with half-lives of 42 and >72 h, respectively. The use of inactive and activated macrophages (RAW 264.7) resulted in half-lives of 21 and 12 h, respectively. Besides the generally low amount of endolysosomal peptides as a consequence of the high protein stability, the most prominent clusters were interestingly observed in the Der p 1 pro-peptide, i.e., residues 41–51 and 52–62 (35). The relevance of these clusters remains unclear because the pro-peptide is typically cleaved off during protein maturation and is therefore not part of the allergen to which humans are exposed. Many *T* cell epitopes of Der p 1 are listed in the IEDB database. However, since the endolysosomal peptide analysis in this study did not include later time points where more peptides would have been generated, overlap with identified *T* cell epitopes remains to be determined.

Der p 2 showed very high resistance to endolysosomal degradation with proteases from JAWS II, A20 and RAW 264.7 cells. Since most of the allergen was still intact after 72 h, calculation of the half-lives was not possible and remains to be determined (35). Der p 2 revealed two early appearing endolysosomal peptide clusters located between 1 and 17 and 60–77 amino acids. These peptide clusters contain *T* cell epitopes located at residues 1–16 and 58–73, which showed *T* cell reactivity in approximately 14% and 25% of *T* cell clones from 2 house dust mite allergic patients, respectively (93). After 24 h of digestion, a limited number of peptides appeared at 22–31 and 101–107, both of which contain weak (mouse) *T* cell epitopes (94–97). To verify that these and other potential clusters are regions containing relevant *T* cell epitopes, analysis of longer endolysosomal digests (>72 h) are required, because the protein was largely intact after 24 h of digestion.

#### Mite group 5 and group 21 allergens

Blo t 5 is a major allergen of tropical mites, found in the gut and feces, with >70% IgE reactivity in allergic individuals (98). Degradation of recombinant Blo t 5 with JAWS II proteases revealed a high susceptibility and no detectable intact protein after 6 h. An N-terminally truncated allergen lacking the 1-18 residue disordered motif (referred to as Blo t 5-short), corresponding to the processed protein found in nature, was more stable and showed increased resistance to endolysosomal degradation (intact protein up to 24 h) (49). The half-life of Blo t 5 short is higher (∼9 h) compared to Blo t 5-long, which is estimated to be less than 2 h (38). Thus, the processing and cleavage of the N-terminally disordered motif provides Blo t 5 with increased stability during endolysosomal processing, which could influence antigenicity (49, 99). Interestingly, mice immunized with Blo t 5-long and Blo t 5-short showed similar IgG responses. However, Blo t 5-short showed reduced levels of IL-4, IL-5 and IFN-γ, while IL-10 was significantly higher in the mouse model (49). Regardless of kinetics, endolysosomal degradation of both Blo t 5 allergens revealed similar peptide clusters at residues 21–36, 39–57, 64–78, 86–98, 101–117 (numbering starting at N-terminally disordered motif). Early in processing, one cluster located at residues 7–20 was identified within the N-terminal disordered motif (49). Using peptide mapping, two mouse *T* cell epitopes within amino acids 59–73 and 84–103 have been identified (100). Both are localized within endolysosomal peptide clusters 67–80 and 89–101, although the observed peptide abundances were moderate (38, 49, 100). The most dominant peptide clusters ranging from 39 to 60 did not overlap with any mouse *T* cell epitope. Further epitope mapping studies using human cells are needed.

Blo t 21 is another major allergen found in the gut and feces of tropical mites. It shares 39% sequence identity with Blo t 5 and co-sensitization is often observed (101, 102). Degradation of Blo t 21-short (deletion of the N-terminally disordered motif) with proteases from JAWS II endolysosomes revealed a half-life of ∼17 h, which is higher compared to Blo t 5 (∼9 h). Two prominent peptide clusters between amino acids 35-52 and 92-113 were observed throughout the digestion period of 48 h, although the number of peptides in the C-terminal cluster decreased with time. Experimentally identified *T* cell epitopes of Blo t 21 are not yet available. However, *in silico* prediction suggests a large *T* cell epitope between residues 92–103, which is within the peptide cluster found by endolysosomal degradation (38).

In addition, two hybrid molecules of Blo t 5 and 21 were generated by fusing identified *T* cell epitopes and excluding the disordered N-terminal motif. BTH1 represents the wild-type sequence, while BTH2 is a hybrid molecule with point mutations for improved protein stability and hypoallergenicity. While no increased thermal stability of BTH2 was observed, this hybrid showed lower IgE binding reactivity compared to Blo t 5 and Blo t 21 as well as the hybrid BTH1. When the hybrids were tested for susceptibility to JAWS II endolysosomal proteases, the half-lives were 39 h for BTH1 and 9 h for BTH2. These results are consistent with the general protein stability measured during thermal denaturation (BTH1 Tm = 64°C, BTH2 Tm = 52°C). With respect to thermal and endolysosomal stability, BTH1 was enhanced while BTH2 was slightly decreased compared to the wild-type allergens (Blo t 5 Tm = 56°C, Blo t 21 Tm = 57°C). BTH2 was immunogenic and induced a shift towards a Th1 response in mice. A comparison of immunogenicity and antigenicity with the wild-type allergens and BTH1 was not possible as these were not evaluated in the mouse model. During endolysosomal degradation, both BTH1 and BTH2 showed peptide clusters at residues 1–17, 19–42, 47–61 and 72–90 (numbering including the N-terminal disordered motif corresponds to residues 18–34, 36–59, 64–78, 89–107). The experimental mouse *T* cell epitope of Blo t 5 was not represented in the identified peptide clusters. However, BTH2 in particular generated many C-terminal peptides that are consistent with a predicted Blo t 21 *T* cell epitope, but those require further investigation (38).

#### Tropomysosins

Tropomyosins from four different species were studied using the endolysosomal degradation assay with proteases from JAWS II cells (37). Tropomyosins are two-stranded alpha-helical, coiled-coil proteins involved in the muscle system of animals. The study investigated proteolytic digestion at pH 5.2 and pH 4.5, as opposed to the more commonly used pH 4.8. Pen m 1 is a major shrimp allergen with a sensitization rate of up to 53% in shrimp-allergic patients (103). The allergen was gradually degraded in the endolysosomal digestion, but stable intermediate protein fragments of sizes between 35 and 38 kDa were observed up to 48 h of digestion. Der p 10 is a minor house dust mite allergen and cross-reacts with crustacean homologs. It shows low IgE binding frequencies between 5% and 18%, but in an African population, this rate was up to 55% in mite allergic patients (104). Endolysosomal degradation showed a high resistance of Der p 10 to proteolysis, with the presence of intact protein even after 48 h. A 38 kDa fragment was present in Der p 10 digest, although in low abundance.

Bla g 7 is a minor cockroach allergen with a sensitization rate of 16% in cockroach allergic patients (105). Degradation of Bla g 7 resulted in progressive proteolysis, but the intact allergen was still detectable after 48 h of degradation and, similar to Pen m 1, stable intermediate protein fragments of 35–38 kDa were observed. Ani s 3 is the tropomyosin of the parasitic herring worm larvae, and high IgE levels due to cross-reactivity can be observed in sensitized patients (106). Endolysosomal degradation of Ani s 3 resulted in complete degradation after 48 h at pH 5.2, while it was still detectable when digested at pH 4.5. Similar to Der p 10, a stable protein intermediate of 38 kDa was observed. Except for Ani s 3, the tropomyosins showed similar stability during thermal processing under neutral or acidic conditions. While Ani s 3 is heat-labile at neutral pH, it gains stability under acidic conditions (pH 5.2). Interestingly, protein stability during endolysosomal degradation appeared to be higher at pH 4.5, in contrast to the observations made with Bet v 1 and variants. Tropomyosins are generally very stable in acidic environments and for optimal comparison (107), proteins that are class 1 food allergens and could sensitize via the gastrointestinal tract should be pre-treated using gastric and intestinal enzymes before endolysosomal digestion.

Consistent with the higher stability of all tropomyosins at pH 4.5 as observed by gel electrophoresis, only a few peptides, mostly localized to the N-terminus of the proteins, were detectable after 24 h of digestion. This confirms that a lower pH stabilizes the studied tropomyosins, leaving more intact protein or stable intermediate large protein fragments and thus producing fewer peptide fragments (37). Degradation of Pen m 1 at pH 5.2 resulted in several peptide clusters, i.e., at residues around 12–27, 30–50, 54–66, 70–90, 135–150 and 154–168. At pH 4.5, a dominant peptide cluster was detectable at residues 12–25, which was present throughout the 24 h digestion. This region appears to represent a relevant *T* cell epitope as it was identified in two independent studies (108, 109). Clusters 54–66 and 136–150 contain Pen m 1_41−60_ and Pen m 1_135−150_ epitopes, respectively (109). In a mouse model, Pen m 1_199−213_ and Pen m 1_244−258_ were identified as *T* cell epitopes, the former corresponding to a human *T* cell epitope (37, 109). Interestingly, no prominent peptide clusters covering this region were observed during endolysosomal degradation.

Endolysosomal degradation of Der p 10 at pH 5.2 resulted in peptide clusters at 14–30, 31–45 and 80–90, while at pH 4.5 a cluster at 12–35 was observed. Degradation of Bla g 7 resulted in peptide clusters at 1–10, 10–25, 30–45, 70–90, 95–105, 115–130, 130–150, 155–170, 170–200. At pH 4.5, a peptide cluster was detected at 10–25. Degradation of Ani s 3 at pH 5.2 produced many peptide clusters spanning almost the entire sequence. Specifically, peptides were aligned at residues 1–10, 10–30, 50–80, 110–130, 135–150, 150–170, 170–200, 205–230, 250–270. Interestingly, Ani s 3 showed a different profile with more peptide clusters generated in the middle and C-terminus of the protein (37). Similar to the digestion of the other tropomyosins at pH 4.5, there was a significant decrease in the number of peptides and only one strong peptide cluster was detected within residues 10–35, which contains a *T* cell epitope of Pen m 1 (108). To date, there are no experimentally verified *T* cell epitopes of Der p 10, Bla g 7, or Ani s 7.

## Conclusions

The endolysosomal degradation assay is a powerful *in vitro* tool to mimic proteolytic processing in the endolysosomal compartment. The straightforward assay allows the determination of endolysosomal stability which is expressed as half-life of a protein under distinct methodological settings. Identified peptide clusters can be considered as *T* cell epitope candidates and thus, in combination with *in silico* MHC II binding studies, narrow down the number of synthetic peptides to be tested in *T* cell epitope mapping studies. This would be particularly relevant in allergy research as experimentally verified *T* cell epitopes are not available for all IgE-binding molecules. In addition, the time-dependent appearance of peptide clusters can be analyzed, providing additional data for predicting protein stability and cleavage accessibility to proteases (58, 110).

A limitation of the endolysosomal degradation assay seems to be the moderate reproducibility of protein degradation kinetics and thus protein stability. For example, the determined half-lives of Bet v 1.0101 ranged from 2 to 14 h, which allows only comparisons of proteins within the same experiment. The source of the observed divergences could be related to different levels of proteolytic enzymes in the analyzed antigen-presenting cells and/or different activity of the endolysosomal protein cocktail. In addition, the protocols are not fully harmonized regarding the ratio of allergen to proteolytic enzymes (5 µg of allergen is digested with 7 or 7.5 µg of proteins from the endolysosomal compartment). Different methods (e.g., colorimetric vs. UV-based methods or amino acid analysis) used for protein quantification could also influence the endolysosomal degradation, which in turn influences the ratio of digestible protein to proteases. The different degradation susceptibility when digested at different pH conditions also indicates the importance of correct buffer settings when performing the assay.

Peptide clusters identified by mass spectrometry-based analysis have traditionally considered the identification of different peptides within a given protein region. This can lead to both over- and under-estimation of clusters, as many slightly different peptides are considered more relevant in graphical representations. In general, there is a need for additional standardization of endolysosomal degradation to allow for assay and laboratory independent comparison of different proteins.

To improve the reproducibility of the assay and to link it to immunogenicity, a standardized endolysosomal degradation assay is being developed within the EU-funded project Allergenicity Prediction Toolbox for Novel Foods (ALLPreT). One of the key points is the *a priori* determination of the proteolytic activity of the endolysosomal cocktail by quantitative digestion of reference proteins (e.g., casein) and/or peptides (e.g., cathepsin substrates). We also plan to validate appropriate protein concentration measurements, determination of half-lives and quantitative peptide readout considering peptide intensities. Within ALLPreT, the endolysosomal degradation of allergens as well as non-allergenic homologues from legumes will be analyzed and linked to immunogenicity data from animal studies and human IgE sensitization. In addition, the influence of the food matrix, heating of the proteins and gastrointestinal pre-digestion will be considered for the first time. In addition to professional antigen-presenting cells, other cells such as neutrophils can become functional APCs (111, 112). Epithelial cells and their endolysosomal proteases are of particular interest as these cells are relevant for allergic sensitization and can act as antigen presenting cells (113, 114). Therefore, ALLPreT aims to compare different antigen presenting cells with respect to their endolysosomal degradation potency, proteolytic content, and activity.

As a general hypothesis, proteins that show moderate to high stability during endolysosomal proteolysis can efficiently induce an immune response. In contrast, unstable proteins that are degraded early and completely within the endolysosomal compartment lack peptides for presentation and thus show low immunogenicity (5). For an efficient immune response, MHC II peptide complexes must be presented in sufficient amounts on the cell surface, which requires high peptide abundance in late endosomes (115). It has been postulated that resistance to degradation in the early endosomes but effective degradation in late endosomes is key for efficient antigen presentation (33). Therefore, proteins with “optimal” stability which are stable in the early endosomes but are efficiently degraded in late endosomes have a higher potential to be presented by APCs. However, hyperstabilized proteins that survive late endosomal degradation result in low *T* cell epitope availability as the majority of protein is still intact, which can lead to abolishment of the immune response (19). It has to be noted that results supporting this hypothesis were mostly obtained from Bet v 1, and it remains to be determined if other allergens are following this concept.

The extent of *T* cell receptor interaction with peptide MHC II complexes influences the differentiation fate of naïve *T* cells (59). In particular, high doses of antigen are thought to promote a Th1 response, whereas low doses favor a Th2 response (41, 44). The extent of peptide presentation also depends on the type and function of the antigen presenting cells. DCs predominantly maintain the long-term survival of peptides for sustained antigen presentation by minimizing premature and rapid protein degradation. This is facilitated by the comparatively low levels and reduced activity of proteases in DCs (5). In general, immune responses in mouse models are influenced by administration routes and adjuvants. When investigating class 1 food allergens, the matrix as well as gastrointestinal (pre)-digestions should be considered when performing the endolysosomal degradation assay.

Currently, *in vitro* endolysosomal studies investigating different pH conditions of allergens are limited to three studies. Machado et al. proposed a mechanism for antigen presentation of Bet v 1 that seems to be most efficient when the antigen is stable in the early endosome (higher pH) followed by sufficient degradation in the late endosome (lower pH) (37). A similar pattern was found for Amb a 1 which showed lower endolysosomal stability at lower pH levels (36). These results suggest that intrinsic protein properties such as pH-dependent protein stability, tertiary structure, and the presence of differentially stable protein domains influence the accessibility of proteolytic cleavage sites during antigen processing. The next level of simulated *in vitro* endolysosomal degradation may integrate these variables and thus provide additional parameters and insights, but further studies using different pH conditions are needed to fully address this issue.

In conclusion, the susceptibility and peptides obtained from the endolysosomal degradation assay are powerful tools for understanding protein immunogenicity and *T* cell reactivity. Systematic analysis and linkage with physicochemical as well as immunological data will enable the establishment of generally applicable tools for use in machine learning that could be useful for predicting protein immunogenicity and allergenicity. This feature could in the future be used for risk assessment of novel foods and in the generation of protein-based immunotherapeutics.
